# Benchmarking performance of an automatic polysomnography scoring system in a population with suspected sleep disorders

**DOI:** 10.3389/fneur.2023.1123935

**Published:** 2023-02-17

**Authors:** Bryan Peide Choo, Yingjuan Mok, Hong Choon Oh, Amiya Patanaik, Kishan Kishan, Animesh Awasthi, Siddharth Biju, Soumya Bhattacharjee, Yvonne Poh, Hang Siang Wong

**Affiliations:** ^1^Health Services Research, Changi General Hospital, Singapore, Singapore; ^2^Department of Respiratory and Critical Care Medicine, Changi General Hospital, Singapore, Singapore; ^3^Department of Sleep Medicine, Surgery and Science, Changi General Hospital, Singapore, Singapore; ^4^Duke-NUS Medical School, Singapore, Singapore; ^5^Centre for Population Health Research and Implementation, SingHealth Office of Regional Health, Singapore, Singapore; ^6^Neurobit Inc., New York, NY, United States; ^7^Department of Biotechnology, Indian Institute of Technology, Kharagpur, India; ^8^National Center for Biological Sciences, Tata Institute of Fundamental Research, Bengaluru, India

**Keywords:** automatic sleep scoring, sleep-disordered breathing, machine learning, AI sleep scoring, sleep staging

## Abstract

**Aim:**

The current gold standard for measuring sleep disorders is polysomnography (PSG), which is manually scored by a sleep technologist. Scoring a PSG is time-consuming and tedious, with substantial inter-rater variability. A deep-learning-based sleep analysis software module can perform autoscoring of PSG. The primary objective of the study is to validate the accuracy and reliability of the autoscoring software. The secondary objective is to measure workflow improvements in terms of time and cost *via* a time motion study.

**Methodology:**

The performance of an automatic PSG scoring software was benchmarked against the performance of two independent sleep technologists on PSG data collected from patients with suspected sleep disorders. The technologists at the hospital clinic and a third-party scoring company scored the PSG records independently. The scores were then compared between the technologists and the automatic scoring system. An observational study was also performed where the time taken for sleep technologists at the hospital clinic to manually score PSGs was tracked, along with the time taken by the automatic scoring software to assess for potential time savings.

**Results:**

Pearson's correlation between the manually scored apnea–hypopnea index (AHI) and the automatically scored AHI was 0.962, demonstrating a near-perfect agreement. The autoscoring system demonstrated similar results in sleep staging. The agreement between automatic staging and manual scoring was higher in terms of accuracy and Cohen's kappa than the agreement between experts. The autoscoring system took an average of 42.7 s to score each record compared with 4,243 s for manual scoring. Following a manual review of the auto scores, an average time savings of 38.6 min per PSG was observed, amounting to 0.25 full-time equivalent (FTE) savings per year.

**Conclusion:**

The findings indicate a potential for a reduction in the burden of manual scoring of PSGs by sleep technologists and may be of operational significance for sleep laboratories in the healthcare setting.

## 1. Introduction

An estimated 50–70 million people in the US have a sleep disorder (1). Economic modeling of five OECD countries estimated the economic loss due to sleep loss to be up to 3% of GDP (2). Polysomnography (PSG) remains the gold standard for measuring sleep. The medical procedure involves concurrent measurement of multiple physiological signals comprising of electroencephalogram (EEG), electrooculogram (EOG), electromyogram (EMG), electrocardiogram (ECG), nasal pressure, airflow, thoracic and abdominal movement, and blood oxygen saturation, among others. Following data collection, the sleep technologist spends ~1–2 h manually scoring the record as per standardized criteria. The most widely adopted criteria are established by the American Academy of Sleep Medicine (AASM), which are updated regularly (3–6).

The process of scoring can be broadly divided into two major tasks: sleep staging and detection of associated respiratory events. Staging involves the division of the record into 30-s epochs and assigning one of the five sleep stages (Wake, N1, N2, N3, and REM) based on patterns in the EEG/EOG/EMG channels. This is followed by identification of various events across different channels. This includes identification of oxygen desaturation events, arousals, apneas, hypopneas, and periodic leg movements. Wherein apneas can be further categorized into obstructive, mixed, and central, and arousals can be categorized into spontaneous, respiratory, or limb movement related. The process is time-consuming and requires strong expertise and consistent attention for reliable results. Although the scoring criteria are standardized, the process remains highly subjective, which introduces significant inter-rater variability (7–11). A meta-analysis of 11 studies found an average agreement for sleep staging at Cohen's kappa of 0.76, indicating substantial agreement (8). The agreement varied greatly across different sleep stages, with the lowest average kappa of 0.24 for N1 and the highest kappa of 0.70 for wake. Although inattention and bias play a role in disagreements, most of the variability in sleep staging is attributed to the fact that many epochs legitimately do not have a clear classification (12). With regard to primary respiratory outcomes like the apnea–hypopnea index (AHI) or oxygen desaturation index (ODI), the agreement among raters is excellent (9). However, the agreement across specific respiratory events can be low (9, 11). Specifically, disagreements between apnea and hypopnea and the types of apneas are common (11).

Therefore, automatic scoring can play a significant role in reducing the burden on sleep technologists while simultaneously reducing variability in scoring. In this study, we benchmark the performance of an automatic scoring system called Neurobit PSG (Neurobit Inc., New York, USA). Neurobit PSG uses deep-learning (DL)-based algorithms to stage sleep and a combination of DL and rule-based systems to identify respiratory events. By default, it scores as per the 2012 AASM standard (3) but is flexible to accommodate popular alternate standards. To establish the viability of such a system, it is important to compare the level of agreement between automatic scoring and experts to an agreement between experts. In addition, productivity gains through the use of the system can also be established.

## 2. Materials and methods

### 2.1. The sleep scoring system—Training dataset

The sleep scoring system called *Neurobit PSG* was developed by Neurobit Inc., New York, NY, USA. The system uses DL-based architecture to provide sleep staging and detection of associated respiratory events. The system was trained and tested on private datasets comprising 12,404 PSG recordings collected at academic sleep centers in South-East Asia (35%), North America (30%), and Europe (30%). In all, 59% of the total assessed participants had a suspected sleep disorder, whereas the remaining 41% of participants were healthy subjects. The mean age of such aggregated dataset was 42.3 ± 16.8 (mean ± std) years. The training data were scored as per the 2007 AASM standards or higher. The software is designed to operate on two EEG channels C3-A2 and C4-A1; two EOG channels E2-A2 and E1-A2; and a bipolar EMG channel for staging. Alternate EOG derivations referenced to A1 or a mix of A1 or A2 are also acceptable by the software. SpO_2_ channel is used for desaturation. Airflow, Pressure, Thoracic, and Abdominal channels are used for the detection of respiratory events. The selection of input channels was made based on common channels available across the complete training dataset. As per the AASM standards (5), the C4-A1 channel and its backup channel C3-A2 are present in both the recommended and alternate EEG derivations. For EOG channels, AASM recommends derivations referenced to A2, but derivation referenced to A1 is also acceptable. The software is designed to automatically handle electrode fall off, noisy, or missing channels.

### 2.2. Subjects

Overnight, in-laboratory PSG recordings from adult patients referred to the clinical sleep laboratory at Changi General Hospital, Singapore, with suspected organic and functional sleep disorders were included in the study. The scoring software was never trained or tested on data from the sleep laboratory before. The study was approved by the Singhealth Centralized Institutional Review Board (CIRB Ref. 2020/2000).

A total of 94 subjects participated in the study. Data from the first five subjects were used to ensure that the software was installed and integrated properly at the clinic. These records were not considered for further analysis. Finally, data from 86 subjects (18 women and 68 men) were included in the comparative analysis. The set was composed of ~67.4% Chinese, 20.9% Malay, 9.3% Indians, and 2.3% other races, which is representative of the Singaporean population. Notably, 31.4% of the subjects had hypertension, 17.4% had diabetes, 1.2% had chronic obstructive pulmonary disease, 25.6% had hyperlipidemia, 4.7% had ischemic heart disease, 10.5% had asthma, and 4.7% had depression. The mean age of the subjects was 44.0 ± 14.4 (range 14–75) years. Further clinical profiling of patients can be found in Table 1.

**Table 1:** Clinical and demographic characteristics of patients in dataset.

**Demographic/clinical characteristics**	**Mean ±SD or *N* (%)**	**Min**	**Max**
Age (years)	44.0 ± 14.4	14	75
Men	68 (79.1)	–	–
Race Chinese Malay Indian Others	58 (67.4) 18 (20.9) 8 (9.3) 2 (2.3)	–	–
BMI (kg/m^2^)	30.9 ± 7.6	18.6	65.4
AHI total (events/h)	35.3 ± 32.6	0.3	134
REM AHI (events/h)	35.2 ± 29.5	0	114.8
NREM AHI (events/h)	34.8 ± 33.7	0.2	135.8
Supine AHI (events/h)	39.8 ± 33.9	0	134
Non supine AHI (events/h)	21.6 ± 31.5	0	138.5
ODI (events/h)	28.1 ± 30.1	0	108.8
% TST SPO_2_ < 90%	9.8 ± 19.7	0	87.4
Arousal index (events/h)	29.5 ± 24.0	4.5	107

### 2.3. Protocol

Recordings were done on a Compumedics (Melbourne, Australia) PSG recorder. Recording signals included EEG channels: C4-A1, C3-A2, F4-A1, F3-A2, O2-A1, and O1-A2; EOG channels: E2-A2 and E1-A2; EMG channels: bipolar EMG, and Left and Right Leg EMG channels; and single ECG channel all sampled at 256 Hz. Respiratory channels, namely, Airflow, Pressure, Thoracic, and Abdominal channels sampled at 32 Hz. Arterial oxyhemoglobin saturation (SpO_2_) was sampled at 16 Hz. The recording montage was based on recommendations of the AASM (3). The signals were stored, viewed, and analyzed using the Compumedics Profusion software version 4.0.

The records were scored manually by a group of trained Registered Polysomnographic Technologist (RPSGT) at the hospital as per the 2012 AASM guidelines (3). Each record was scored by one of the five technologists. For the sake of simplicity, we refer to the group of technologists as “**expert 1**.” The scoring was done visually within the Compumedics Profusion software. Specifically, for sleep staging, 30-s epochs were assigned one of the five stages (Wake, N1, N2, N3, REM) based on patterns in the EEG/EOG/chin EMG channels. Apneas were identified if there was a 90% or more reduction in airflow for at least 10 s compared with the baseline. For hypopnea, the criteria were set as a drop in thoracoabdominal movement or airflow drop of 30% or more compared with baseline for at least 10 s with at least 3% desaturation or an associated EEG arousal. Arousals were scored if there was an abrupt shift in EEG power lasting at least 3 s. The raw PSG data were also exported to European Data Format (EDF) (13), an open standard for the exchange of physiological data. The records were anonymized during export. These exported data were then securely sent to a third-party independent scoring company MBS Sleep Scoring Services, LLC (St. Louis, MI, USA) where it was scored by an RPSGT (**expert 2**). The technologist used the Philips Sleepware G3 (Philips Respironics, Inc., PA, USA) software to view and score the data manually as per the same 2012 AASM standards (3).

For automatic scoring (**auto**), an on-premise version of Neurobit PSG was installed at the hospital. This was necessary because the hospital did not have access to the internet in compliance with local security guidelines. The exported EDF files were transferred to the local machine using a secure thumb drive where they were auto-scored, and the results were generated in a Profusion compatible XML format. The scores were then transferred back to the Profusion software for review by the technologists. The local version of Neurobit PSG was installed in a *headless* mode, i.e., there was no user interface. As soon as EDF files were placed in a designated folder, the scoring started automatically based on the preconfigured montage, and the results were generated in the same folder. The only visual indication provided was an external LED cube that flashed when scoring was in progress.

#### 2.3.1. Manual review of automatic scores

Automated scoring systems are expected to be used in conjunction with expert review to achieve high levels of reliability. Based on the performance and limitations of the automatic system, a manual review can be optimized to ensure excellent reliability while minimizing the time required to review. To avoid introducing systematic bias into the review process, experts from the clinic (expert 1) were instructed to review the automatic scores thoroughly. The reviewed scores (**review**) were then compared with automatic scores as well as the other expert scores to come up with an optimal strategy to maximize the throughput of scoring while maintaining a high degree of scoring accuracy and reliability. The time taken by expert 1 to manually score as well as to review the automatic scores was also logged.

#### 2.3.2. Time motion study

Hired research assistants (RAs) were deployed at the sleep laboratory to observe and track the time spent explicitly by sleep technologists (expert 1) in order to complete the manual scoring of sampled PSGs. The RAs made sure that their presence did not distract the technologists in their day-to-day activities. RAs were required to differentiate tasks undertaken by the sleep technologists (expert 1) according to whether these tasks were related to scoring of PSGs (e.g., answering phone calls, going to toilets, and addressing queries by colleagues). The same set of sampled PSGs was then automatically scored by the automated scoring system (auto), and the amount of time spent autoscoring every PSG along with the additional time spent by the sleep technician to manually review the auto-scored PSGs were tracked by the RAs.

### 2.4. Data analysis

#### 2.4.1. Statistical analysis

In the presence of significant variability in scoring between experts, it is difficult to determine what should be considered the ground truth. Instead of comparing the automatic scores with a single expert, it is, therefore, important to compare the automatic scores with multiple experts and stack them against the agreement between the experts. Ideally, the agreement between automatic and experts should be indistinguishable from an agreement between experts. In the study, we use accuracy, Cohen's kappa (κ) and intra-class correlation coefficient (ICC) to compare scores between experts and automatic scoring. Accuracy quantifies the agreement between two raters, but it has been used as a simple measure of inter-rater reliability (IRR) (14). Cohen's kappa and ICC are appropriate measures of IRR. The IRR between experts provides an upper bound for the automatic scoring performance.

#### 2.4.2. Sleep staging performance

For comparing sleep stages, an epoch-by-epoch comparison was carried out between auto, expert 1, and expert 2. This was done by combining all epochs across all subjects. A confusion matrix was calculated along with overall accuracy and Cohen's kappa. Cohen's kappa is considered to be a robust measure for IRR as it accounts for agreement due to random chance (14). The kappa statistic varies between −1 and 1, with values appaindicating no agreement and 0.01–0.20 as none to slight agreement, 0.21–0.40 as fair, 0.41–0.60 as moderate, 0.61–0.80 as substantial, and 0.81–1.00 as almost perfect agreement. To quantify the stage-specific agreement, kappa was also computed for each stage across expert–expert and expert–auto comparisons. A similar comparison was also carried out between auto and the concordance between the two experts. This was computed by only considering epochs where both the experts agreed with each other. To carry out the statistical comparison, subject-wise accuracy and kappa were also calculated. A one-way repeated-measures ANOVA was carried out to test if mean agreement between experts and between expert and auto was statistically different.

In addition to epoch-by-epoch comparison, derived sleep measurements were also compared between experts and auto. Specifically, total sleep time (TST), sleep efficiency, and time spent (both absolute and percentage of TST) in N1, N2, N3, REM, sleep latency, and REM latency were derived for every subject for each rater. Agreement between the raters was accessed through ICC (15). Based on the 95% confidence interval (CI) of the ICC estimate, values <0.5, between 0.5 and 0.75, between 0.75 and 0.9, and >0.90 are indicative of poor, moderate, good, and excellent reliability, respectively (15).

#### 2.4.3. Detection of respiratory events

Respiratory events included apneas, hypopneas, arousals, and oxygen desaturation. Apneas were further subcategorized into central, mixed, and obstructive apneas. For each subject, the number of events were counted and compared across raters. Agreement between the raters was accessed through ICC (15). A comparison was also carried out between the auto and the average of the two experts. Indices were calculated by dividing the event count by TST. This included AHI and ODI. For AHI and ODI, which are the primary respiratory outcomes, Pearson's correlation with expert estimates was also obtained.

#### 2.4.4. Time motion study

Manual scoring of trained experts at the hospital (**expert 1**) was compared against automatic scoring coupled with the additional time taken by the sleep technologists to manually review the automated scores generated using the software (**auto**). The paired *t*-test was used to assess the time difference between manual and automated scoring. The time saved with the use of automated scoring was used to estimate manpower FTE savings with the use of automated sleep scoring in our healthcare setting.

#### 2.4.5. Sample size calculation

The anticipated mean timing for manual scoring of a PSG is 45 min (SD = 10) and automated scoring at 40 min. Assuming α (type 1 error) = 0.05, β (type 2 error) = 0.9, and a group allocation ratio of 1:1, the sample size required for this analysis would be 168 patients, with 84 patients in the manual and autoscoring groups each. As both the manual and autoscoring were performed on the same patient, the sample size required would be halved at 84 patients.

## 3. Results

**Figure 1:**
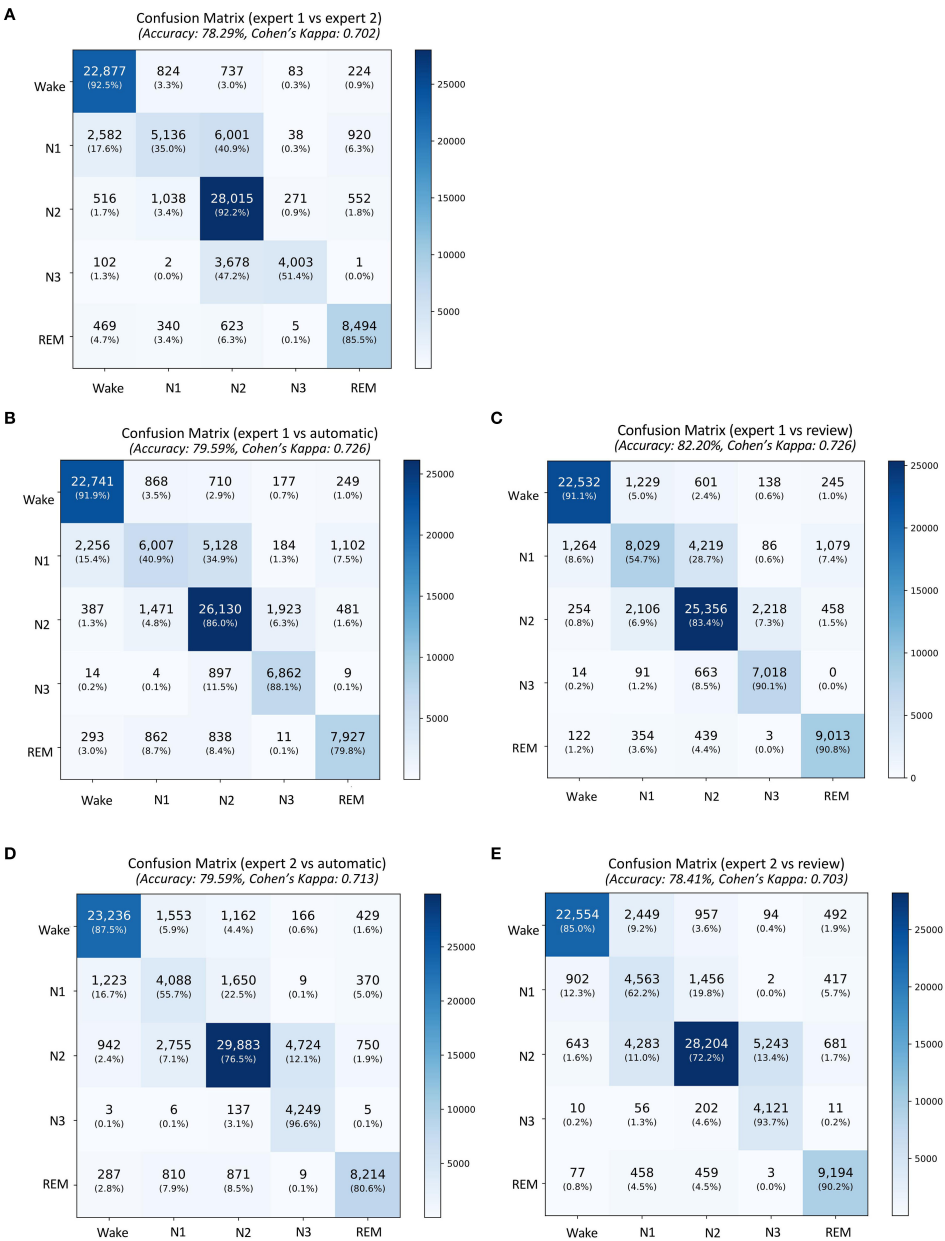
Confusion matrix comparing sleep stages between **(A)** the expert 1 (rows) and expert 2 (columns), **(B)** expert 1 and automatic staging, **(C)** expert 1 and review of the automatic scores by expert 1, **(D)** expert 2 and automatic staging, and **(E)** expert 2 and review of the automatic scores by expert 1. The confusion table is constructed by combining sleep stages across all subjects.

**Figure 2:**
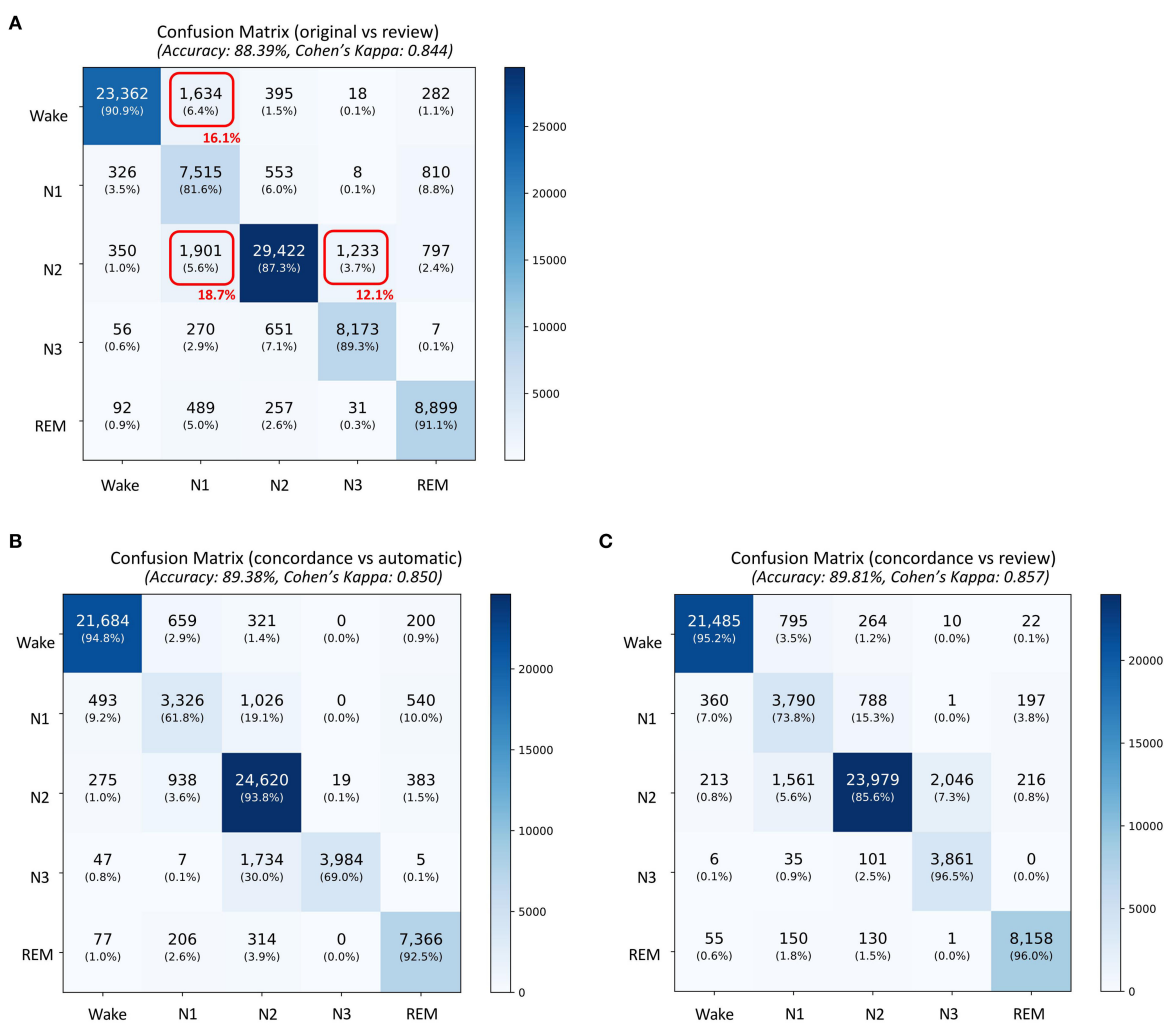
Confusion matrix comparing sleep stages between **(A)** the original scores generated by automatic algorithm (rows) and automatic scores obtained after review (columns), **(B)** concordance of the two experts and automatic staging (for concordant epochs), and **(C)** concordance of the two experts and automatic staging after review (for concordant epochs. The confusion table is constructed by combining sleep stages across all subjects. The red boxes in **(A)** indicate the three largest changes made after review. The numbers in red indicate percentage of total changes made during the review.

A total of 87,531 epochs (729.4 h) were compared between the experts and the auto. The confusion matrix is presented in Figure 1. The overall agreement between the two experts (Figure 1A) was 78.29%, with κ of 0.702 indicating substantial agreement. The overall agreement between expert 1 and auto (Figure 1B) was 79.59% with κ 0.726 and between expert 2 and auto (Figure 1D) was 79.59% with κ 0.713, again indicating substantial agreement. In absolute terms, the agreement between auto and the experts was higher than between both the experts for both accuracy and kappa. For individual sleep stages, the results are summarized in Table 2. The agreement was highest for wake (κ = 0.847 between experts vs. κ = 0.853 between expert and auto), followed by REM (κ = 0.824 between experts vs. κ = 0.790 between expert and auto), N2 (κ = 0.683 between experts vs. κ = 0.703 between expert and auto), N3 (κ = 0.633 between experts vs. κ = 0.695 between expert and auto), and finally N1 (κ = 0.399 between experts vs. κ = 0.436 between expert and auto). Agreement between auto and the concordance of the two experts was 89.38%, with κ 0.850 indicating almost perfect agreement (Figure 2B). Between the two experts, the highest agreement was obtained for wake and REM sleep, while N3 and N1 sleep showed the least agreement, respectively (Table 2). A similar trend was also observed between expert and auto scores.

**Table 2:** Table comparing stage-wise agreement in terms of Cohen's kappa between experts and between each expert and automatic scoring.

	**Wake**	**N1**	**N2**	**N3**	**REM**
Expert1 vs. expert 2	0.847	0.399	0.683	0.633	0.824
Expert 1 vs. automatic	0.862	0.429	0.709	0.790	0.780
Expert2 vs. automatic	0.843	0.442	0.696	0.600	0.800
Expert vs. automatic (mean)	0.853	0.436	0.703	0.695	0.790

The mean agreement between automatic scoring and the two experts are computed in the final row.

**Figure 3:**
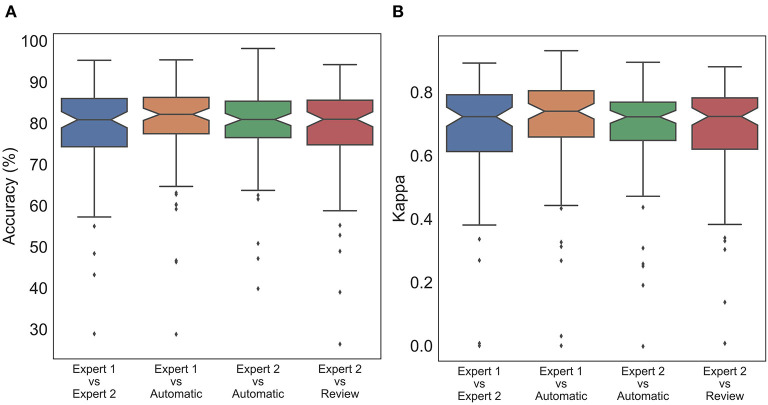
Box plot of subject wise **(A)** accuracy and **(B)** Cohen's Kappa comparing staging between experts and automatic scoring. No statistically significant difference was found between expert scores and automatic scores for both accuracy and Cohen's kappa.

To statistically compare the staging agreement, subject-wise accuracy and kappa were plotted (Figure 3). The box plot indicated a few outliers for which the accuracy and corresponding kappa were very low. It was also observed that few records had very low kappa while still having high accuracy. On closer inspection, these records were dominated by one or two stages, while other stages were completely missing. In such scenarios, kappa measure can be low, even when accuracy is high. The average and aggregate accuracy and kappa are presented in Table 3. The average agreement between the two experts was 78.08 ± 11.70% with κ of 0.673 ± 0.172. Agreement between expert 1 and expert 2 with auto were 79.38 ± 11.08% with κ 0.695 ± 0.172 and 79.52 ± 9.82% with κ of 0.680 ± 0.158. Similar to the combined agreements, subject-wise average accuracy and kappa were higher between auto and experts as compared with between the experts. However, a one-way repeated-measures ANOVA did not detect any statistically significant difference between the experts as compared with between auto and experts for both accuracy $F_{170}^{2}=1.471,\;P=0.233$ and kappa $F_{170}^{2}=1.626,\;P=\;0.200$.

**Table 3:** Table comparing accuracy and Cohen's kappa between subject wise measures and combined sleep stages.

	**Accuracy**		**Kappa**	
	**Average**	**Combined**	**Average**	**Combined**
Expert 1 vs. expert 2	78.08% ± 11.70%	78.29%	0.673 ± 0.172	0.702
Expert 1 vs. automatic	79.38% ± 11.08%	79.59%	0.695 ± 0.172	0.726
Expert 2 vs. automatic	79.52% ± 9.82%	79.59%	0.680 ± 0.158	0.713
Expert 1 vs. review	81.91% ± 13.34%	82.20%	0.740 ± 0.187	0.726
Expert 2 vs. review	78.20% ± 11.66%	78.41%	0.671 ± 0.179	0.703

Mean and standard deviations are shown for subject wise measures.

For derived sleep measures, except for N2%, all other measures showed good to excellent ICC between the average of the two raters and auto (Table 4). In fact, except for latency, the agreement between auto and mean of the two raters was higher than that of agreement between the two raters. Between the two experts, agreement remained good to excellent for most measures, except for N1 time (ICC 0.594) and N2% (ICC 0.607). The overall agreement along with the 95% CI is presented in Table 4A.

Table 4Intraclass correlation coefficient (ICC) **(A)** of various derived sleep measures compared against each pair of scorers, **(B)** between experts and average of the experts and automatic scores after review.

**Table:** 

**A**.				
**Measurement**	**Expert 1 vs. expert 2**	**Average vs. automatic**	**Expert 1 vs. automatic**	**Expert 2 vs. automatic**
TST	0.873 (0.810 ± 0.920)	**0.945 (0.920** **±0.960)**	0.904 (0.860 ± 0.940)	0.927 (0.890 ± 0.950)
Efficiency	0.844 (0.770 ± 0.900)	**0.935 (0.900** **±0.960)**	0.879 (0.820 ± 0.920)	0.920 (0.880 ± 0.950)
N1 time	0.594 (0.440 ± 0.710)	**0.827 (0.750** **±0.880)**	0.687 (0.560 ± 0.780)	0.796(0.700 ± 0.860)
N2 time	0.806 (0.720 ± 0.870)	**0.829 (0.750** **±0.880)**	0.764 (0.660 ± 0.840)	0.806 (0.720 ± 0.870)
N3 time	0.756 (0.650 ± 0.830)	**0.857 (0.790** **±0.900)**	0.855 (0.790 ± 0.900)	0.766 (0.660 ± 0.840)
REM time	0.793 (0.700 ± 0.860)	**0.873 (0.810** **±0.920)**	0.775 (0.670 ± 0.850)	0.883 (0.830 ± 0.920)
N1%	0.753 (0.640 ± 0.830)	**0.896 (0.840** **±0.930)**	0.807 (0.720 ± 0.870)	0.872 (0.810 ± 0.910)
N2%	0.607 (0.450 ± 0.730)	**0.713 (0.590** **±0.800)**	0.539 (0.370 ± 0.670)	0.732(0.620 ± 0.820)
N3%	0.763 (0.660 ± 0.840)	**0.837 (0.760** **±0.890)**	0.837 (0.760 ± 0.890)	0.746 (0.640 ± 0.830)
REM%	0.782 (0.680 ± 0.850)	**0.829 (0.750** **±0.890)**	0.700 (0.570 ± 0.790)	0.869 (0.810 ± 0.910)
Latency	**0.972 (0.960** **±0.980)**	0.911 (0.870 ± 0.940)	0.920 (0.880 ± 0.950)	0.889 (0.830 ± 0.930)
REM latency	**0.963 (0.940** **±0.980)**	0.863 (0.800 ± 0.910)	0.867 (0.800 ± 0.910)	0.843 (0.770 ± 0.900)

**Table:** 

**B**.		
**Measurement**	**Expert 1 vs. expert 2**	**Average vs. review**
**TST**	0.873 (0.810 ± 0.920)	**0.951 (0.930** **±0.970)**
**Efficiency**	0.844 (0.770 ± 0.900)	**0.940 (0.910** **±0.960)**
**N1 time**	0.594 (0.440 ± 0.710)	**0.831 (0.750** **±0.890)**
**N2 time**	0.806 (0.720 ± 0.870)	**0.853 (0.780** **±0.900)**
**N3 time**	0.756 (0.650 ± 0.830)	**0.826 (0.740** **±0.880)**
**REM time**	0.793 (0.700 ± 0.860)	**0.902 (0.850** **±0.930)**
**N1%**	0.753 (0.640 ± 0.830)	**0.886 (0.830** **±0.920)**
**N2%**	0.607 (0.450 ± 0.730)	**0.703 (0.580** **±0.800)**
**N3%**	0.763 (0.660 ± 0.840)	**0.815 (0.730** **±0.880)**
REM%	0.782 (0.680 ± 0.850)	**0.878 (0.820** **±0.920)**
Latency	**0.972 (0.960** **±0.980)**	0.967 (0.950 ± 0.980)
REM latency	0.963 (0.940 ± 0.980)	0.963 (0.940 ± 0.980)

Numbers in brackets indicate 95% confidence interval. When comparing ICC between **(A)** experts and automatic vs. average and **(B)** the experts and automatic against the average after review, bold figures indicate better performance.

For the primary respiratory outcomes including AHI and ODI, the agreement was excellent between raters as well as between raters and auto. For AHI, the ICC between the average of the two raters and auto was 0.958, indicating a near-perfect agreement. The agreement between the experts was lower at ICC 0.902, but was still close to excellent. A similar trend was also observed for ODI (Table 5).

Table 5Intraclass correlation coefficient (ICC) of **(A)** important respiratory indices compared against each pair of scorers, **(B)** automatic scores before and after review as compared with average of the two experts.

**Table:** 

**A**.				
	**Expert 1 vs. expert 2**	**Average vs. automatic**	**Expert 1 vs. automatic**	**Expert 2 vs. automatic**
**AHI**	0.902 (0.850 ± 0.930)	**0.958 (0.940** **±0.970)**	0.972 (0.960 ± 0.980)	0.891 (0.840 ± 0.930)
**ODI**	0.870 (0.810 ± 0.910)	**0.986 (0.980** **±0.990)**	0.957 (0.930 ± 0.970)	0.948 (0.920 ± 0.970)

**Table:** 

**B**.		
	**Average vs. automatic**	**Average vs. review**
**AHI**	0.958 (0.940 ± 0.970)	**0.962 (0.940** **±0.980)**
**ODI**	**0.986 (0.980** **±0.990)**	0.934 (0.900 ± 0.960)

Numbers in brackets indicate 95% confidence interval. When comparing ICC between (A) experts and automatic vs. average, and (B) automatic against the average, before and after review, bold figures indicate better performance.

With regard to the individual respiratory events, the agreement for apneas was good across the board. ICC between the experts was 0.880, while ICC between the average of the two experts and auto was 0.813. When individual apneas were subcategorized, the agreement dropped substantially. Between the experts as well as between auto and average of the two experts, agreement was good for obstructive apnea, moderate for mixed apnea, and poor for central apnea. For hypopneas, agreement between auto and average of the two experts was poor, although the agreement between experts was good. A similar result was also observed for arousals. For desaturation events, the agreement remains excellent for all raters and between auto and raters. The results are summarized in Table 6A.

Table 6Intraclass correlation coefficient (ICC) of (A) various respiratory counts compared against each pair of scorers, (B) automatic scores before and after review as compared with average of the two experts.

**Table:** 

**A**.				
	**Expert 1 vs. expert 2**	**Average vs. automatic**	**Expert 1 vs. automatic**	**Expert 2 vs. automatic**
Apneas	**0.880 (0.820** **±0.920)**	0.813 (0.730 ± 0.870)	0.743 (0.630 ± 0.820)	0.767 (0.660 ± 0.840)
Central apneas	**0.453 (0.270** **±0.610)**	0.390 (0.200 ± 0.560)	0.522 (0.350 ± 0.660)	0.496 (0.320 ± 0.640)
Obstructive apneas	**0.780 (0.680** **±0.850)**	0.753 (0.640 ± 0.830)	0.689 (0.560 ± 0.790)	0.720 (0.600 ±0.810)
Mixed apneas	**0.812 (0.730** **±0.870)**	0.637 (0.490 ± 0.750)	0.663 (0.530 ± 0.770)	0.658 (0.520 ± 0.760)
Hypopneas	**0.846 (0.770** **±0.900)**	0.307 (0.100 ± 0.490)	0.332 (0.130 ± 0.510)	0.665 (0.530 ±0.770)
Arousals	**0.778 (0.680** **±0.850)**	0.458 (0.270 ± 0.610)	0.314 (0.110 ± 0.490)	0.742 (0.630 ± 0.820)
Desaturations	0.987 (0.980 ± 0.990)	**0.996 (0.990** **±1.000)**	0.993 (0.990 ±1.000)	0.970 (0.950 ± 0.980)

**Table:** 

**B**.		
	**Average vs. automatic**	**Average vs. review**
Apneas	0.813 (0.730 ±0.870)	**0.833 (0.750** **±0.890)**
Central apneas	0.390 (0.200 ± 0.560)	**0.655 (0.520** **±0.760)**
Obstructive apneas	0.753 (0.640 ±0.830)	**0.823 (0.740** **±0.880)**
Mixed apneas	0.637 (0.490 ± 0.750)	**0.667 (0.530** **±0.770)**
Hypopneas	0.307 (0.100 ± 0.490)	**0.622 (0.470** **±0.740)**
Arousals	0.458 (0.270 ±0.610)	**0.889 (0.830** **±0.930)**
Desaturations	**0.996 (0.990** **±1.000)**	0.976 (0.960 ± 0.980)

Numbers in brackets indicate 95% confidence interval. When comparing ICC between (A) experts and automatic vs. average and (B) automatic against the average, before and after review, bold figures indicate better performance.

**Figure 4:**
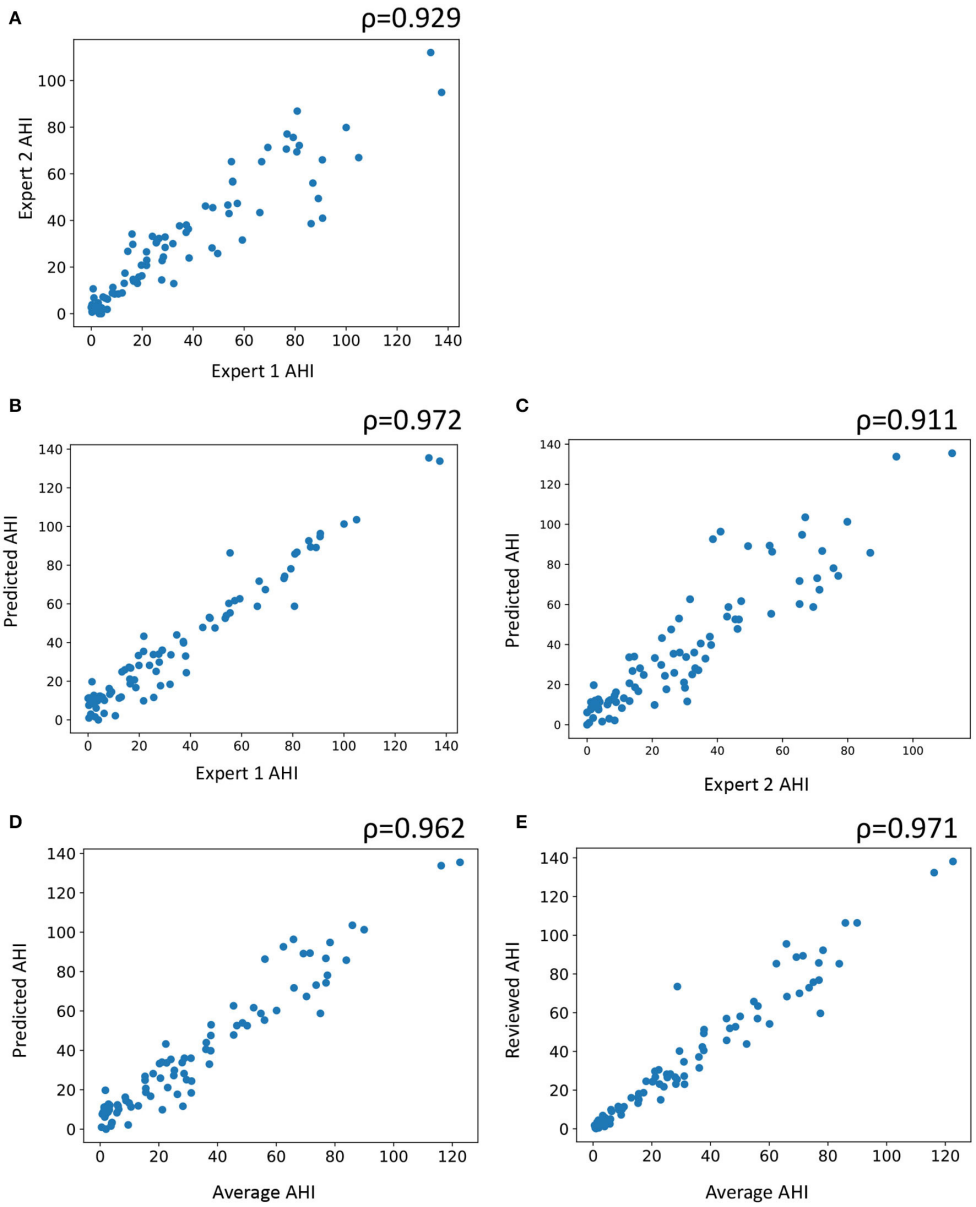
Scatter plot of subject wise apnea-hypopnea index (AHI) estimated between **(A)** the two experts, **(B)** automatic and expert 1, **(C)** automatic staging and expert 2, **(D)** automatic staging and average of both experts, and **(E)** automatic staging after review and average of the two experts. AHI is computed by counting the number of apneas and hypopneas and dividing it by the total sleep time. ρ indicates Pearson's correlation.

For AHI, which is the primary diagnostic criteria for sleep apnea, a scatter plot was obtained between experts and auto as well as between the mean of the two experts and auto (Figure 4). Pearson's correlation between the average of the two experts and auto was 0.972, indicating a near-perfect correlation. The correlation between the experts was also high at 0.929.

### 3.1. Manual review of automatic scores

The auto scores were thoroughly reviewed by expert 1. Following the review, the scores were compared with both experts. Since the review was done by expert 1, it is expected that the agreement of the reviewed scores would match better with that of expert 1. The agreement between expert 1 and automatic increased from 79.59 to 82.2% after the review (Figures 1B, C). Interestingly, Cohen's kappa remained completely unaffected by the review. Agreement with expert 2 dropped after the review from 79.59 to 78.41% (Figures 1D, E). The kappa also reduced from 0.713 to 0.703 following the review. To evaluate which epochs were affected following the review, the confusion matrix between the original auto scores and review was plotted (Figure 2A). A total of 10,160 epochs (11.6% of all epochs) were changed after the review. The three major changes were 1,901 epochs changed from N2 to N1, 1,634 epochs changed from Wake to N1, and 1,233 epochs changed from N2 to N3.

The agreement between the concordance of the two experts and automatic was excellent at 89.38%, with a kappa of 0.850 (Figure 2B). This only increased marginally after the review (89.81%, kappa 0.857) (Figure 2C). With regard to derived sleep measures, ICC for REM latency improved from 0.911 to 0.967 for latency and 0.863 to 0.963 for REM latency (Table 4B).

With regard to primary respiratory outcomes, ICC improved from 0.958 to 0.962 for AHI but declined for ODI from 0.986 to 0.934 following the review (Table 5B). For the individual respiratory events, we saw an improvement in ICC across the board, except for desaturation (Table 6B).

To mitigate any impact of memorization on the review process, the review process was separated from the scoring process by at least 6 months. Furthermore, the scoring and review were randomly assigned to a given technologist based on operational constraints. This further reduces the chances that the same technician scores and reviews the same record.

### 3.2. Time motion study and productivity gains

Manual scoring by **expert 1** took an average of 4,243 s (70.7 min). Automatic scoring took an average of 42.7 s per record. The time taken for autoscoring coupled with a thorough review of the scores by **expert 1** took an average of 1,929 s (32.1 min), representing average time savings of 2,314 s (38.6 min) per patient PSG report generated (*p* < 0.001). With an estimated saving of 38.6 min per patient PSG report, total savings amounts to 28,950 min per year (482.5 h) or a total of 0.25 FTE savings per year based on an estimated load of 750 patients per year requiring sleep disorder-related investigations at an acute care institution in Singapore and FTE of one nurse being equivalent to 1,940.4 h per annum. This is equivalent to 0.33 FTE per 1,000 patients-year.

## 4. Discussion

The AASM in a recent position statement stated that PSG is well-suited for analysis using AI and has the potential to improve sleep laboratory efficiency and yield greater clinical insights (16). The position statement comes in light of recent advances in machine learning (ML) algorithms, specifically DL-based algorithms that have demonstrated phenomenal performance improvements across the spectrum of applications (17). DL algorithms train models directly from data without relying on hand-engineered features or rules (18). As per the AASM, the goal of AI should be to augment expert evaluation of sleep data. While accuracy and reliability are important considerations for such an AI, there are other considerations that are equally important, including logistical, security, ethical, and legal. Commercial systems must address all these considerations before they are allowed to be marketed by the regulators. It is not surprising that despite the strong interest in AI-based sleep scoring within the academic field (19, 20), only a handful of commercial AI scoring solution exists in the market that fully exploits these recent advances in AI. It is, therefore, important to benchmark the performance of such commercial systems as they potentially have a huge impact on clinical practice.

In the present study, we benchmarked the performance of an automatic sleep scoring system called Neurobit PSG on patients referred to a sleep lab with a suspected sleep disorder. The software is trained on large and highly diverse PSG datasets with a good mix of healthy and patient population. To establish a baseline for the scoring, we scored the records independently by two different sets of scorers. Expert 1 was a set of expert sleep technologists at the sleep lab, while expert 2 was a single RPSGT at a commercial scoring company. To avoid any systematic bias in the training of the experts, we ensured that the two sets of experts were geographically isolated. We demonstrated a high degree of concordance between the automatic system and expert scorers. With regard to sleep staging, at an epoch-by-epoch level, the agreement between automatic and experts was consistently higher than the agreement between the two experts. However, no statistically significant difference was observed between expert and manual scoring. With regard to key derived sleep measurements, including TST, sleep efficiency, time spent in various sleep stages, WASO, and latency, the agreement between auto and the experts was excellent. For most measures, there was a higher agreement between auto and the experts than between the two experts. There was excellent agreement between auto and the experts for primary respiratory indices, including AHI and ODI. However, agreement for individual respiratory events can improve.

A thorough review of the scores (including sleep stages and respiratory events) was carried out by expert 1. It was observed that a thorough review of staging introduced bias into the scores and had a negligible or negative impact on agreement. A similar trend was observed for oxygen desaturation events. For the primary respiratory outcome, agreement for AHI improved while that for ODI reduced. For individual respiratory events, we observed significant improvement in agreement following the review. Based on these observations, an optimal review strategy is proposed.

In the following sections, we discuss these results in greater detail.

### 4.1. Sleep staging performance

The overall agreement between the two experts was 78.29%, with κ = 0.702 when epochs across all subjects were combined. This is in line with the previously reported IRR for subjects with suspected sleep disorders. In a study comparing IRR between experienced scorers from eight European sleep laboratories within a large sample of patients with various sleep disorders (7), the overall level of agreement was found to be 76.8%, with κ = 0.682. The authors observed that the IRR varied significantly across different disease conditions. For individual sleep stages, the highest agreement was for REM, followed by wake, N3, N2, and N1. The study relied on an outdated standard for sleep scoring rules published by Rechtschaffen and Kales (21). The R&K standard has significant limitations (22) and has been superseded by the modern AASM standards. A more recent study (9) was carried out involving experts from nine center members of the Sleep Apnea Genetics International Consortium (SAGIC) to establish IRR across international sleep centers. The scoring was done on 15 previously recorded PSGs as per the AASM guidelines. The overall agreement across all epochs was found to be κ = 0.63 (95% CI 0.62–0.63), indicating substantial agreement. Agreement for REM and wake was similar (κ = 0.78), followed by N3 (κ = 0.67), N2 (κ = 0.6), and finally N1 (κ = 0.31). In another study, inter-lab reliability between US and Chinese sleep centers was accessed. Five doctors from China and two doctors from the USA scored 40 overnight PSG records as per the AASM standard. The overall agreement was observed at κ = 0.75. Agreement was highest for REM and wake and lowest for N1. To quantify and improve IRR, the AASM started an inter-scorer reliability program (11). A small dataset comprising 9 record fragments (1,800 epochs) was scored by more than 2,500 scorers, most with 3 or more years of experience. The overall agreement across all epochs and scorers was 82.6%. Again, agreement was highest for REM (90.5%), followed very closely by N2 and wake (85.2 and 84.1%, respectively). Agreement was lower for N3 (67.4%) and lowest for N1 (63%). Unfortunately, Cohen's kappa statistic was not provided in the study.

The IRR between the independent experts provided us with a benchmark to compare against for automatic staging. We observed that the staging performance of the automatic system was similar to that of experts and consistent with prior findings. A stage-wise comparison was also consistent with prior observations, with the highest agreement being for wake, followed by REM, N2, N3, and N1. Both subject level and combined agreement were higher between auto and the expert as compared with between the experts. This was also reflected in the derived measures like TST, WASO, and time spent in individual stages. This is not surprising as most of the derived measures are directly linked to epoch-by-epoch accuracy. The only derived measure where experts had a better agreement compared with between expert and auto was latency. This is interesting as a single incorrectly identified sleep epoch can widely affect latency measurement, even if, at a statistical level, epoch-by-epoch staging accuracy of an automatic system might be indistinguishable from experts. ICC for auto vs. experts was good to excellent (ICC 0.945 for TST and ICC 0.863 for REM latency), there is value in the expert spending some time cross-checking the first sleep and REM stages detected by the autoscoring system. The AASM inter-scorer reliability program also observed that one of the epochs of highest disagreement was REM after N2 (10). We discuss data-driven recommendations for expert review in a later section.

### 4.2. Scoring of respiratory events

The IRR for detecting respiratory events is not as extensively studied as IRR for sleep staging. For respiratory events, the exact location and duration of the events are of less value. Instead, clinical outcomes are associated with the count of such events, sometimes normalized by the TST in the form of indices. Therefore, IRR for respiratory events is evaluated by comparing the event counts/indices across scorers. The AHI and ODI are two such primary outcome measures of sleep-disordered breathing. ICC is usually the metric used to quantify reliability.

IRR for respiratory events is strongly dependent on the rules and specifications. For instance, IRR across three sleep technologists at one centralized scoring center was excellent when the respiratory events were associated with a desaturation event, rather than the presence of an associated EEG arousal (23). This is not surprising as IRR for EEG arousals is usually low to moderate (9, 24, 25). Despite this, the AASM introduced a major change in the definition of hypopnea in 2012. Compared to the 2007 standard (6) where hypopneas were associated with a ≥ 4% drop in oxygen desaturation, the 2012 standard (3) required the presence of ≥ 3% drop in oxygen desaturation and/or an associated EEG arousal. It was observed that the 2012 standard almost always resulted in a higher number of hypopnea events (26). Despite a potential reduction in IRR, the 2012 definition is clinically more relevant (27). Therefore, in the present analysis, we scored the studies based on the updated 2012 definition of hypopnea.

Prior work has shown IRR to be excellent for primary respiratory outcomes, even though agreement can vary widely for individual respiratory events. Within the SAGIC study, Magalang et al. tried to assess the respiratory IRR in addition to staging agreements among the international sleep centers. For AHI, they observed an ICC of 0.95 (95% CI: 0.91–0.98) and for ODI, ICC was 0.97 (95% CI 0.93–0.99), indicating excellent agreement across raters. For individual events, the ICC was lower. ICC was 0.73 (95% CI 0.55–0.88) for total apneas and 0.80 (95% CI 0.65–0.91) for total hypopneas. Subcategorizing apneas further reduced the agreements: 0.70 for obstructive, 0.46 for central, and 0.42 for mixed. In another study, 28 determined the inter-site agreement in respiratory events. They scored a set of 70 records by 10 technologists from five different sleep centers, using three different hypopnea criteria described in the 2007 AASM standards. For AHI, the across-site ICC was excellent at 0.984 (95% CI 0.977–0.990). Across-site ICC for obstructive apnea was 0.861 (95% CI 0.837–0.881) and for central apnea was 0.683 (95% CI 0.640–0.722). For hypopneas, as per the 2007 recommended definitions, the inter-site ICC was 0.843 (95% CI 0.820–0.870). It must be noted that both studies used the conservative definition of hypopnea and oxygen desaturation in the 2007 recommendations. In addition, the second study marked mixed apneas as obstructive (28). Therefore, their results might be inflated compared with scoring using the 2012 rules.

The AASM inter-scorer reliability program also explored respiratory events (11). The sample included 15 monthly records with 200 epochs each. These were scored by over 3,500 scorers. Instead of identifying the location and count of the events, the scorers had to identify if a particular event happened in a shown epoch. Therefore, although the outcome gave an indication of agreement, it is not directly translatable to the actual detection of respiratory events which relies on the count of such events. Nonetheless, within this framework, the correct event type was designated as the majority score and the percentage agreement was used as a proxy for IRR. Overall, 3,000 epochs were included in the analysis, of which 364 (12%) were scored to have a respiratory event by the majority. Out of 364, 172 were hypopnea, 150 were obstructive apnea, 41 were central apnea, and only 1 was mixed apnea. For hypopnea, agreement was 65.4%, obstructive apnea was 77.1%, central was 52.4%, and mixed apnea was 39.8%. The overall agreement for detecting any respiratory event was 88.4%. In other words, while the overall agreement was very good, disagreements in scoring apnea vs. hypopnea and type of apnea were common.

In this study, we observed excellent agreement between automatic and expert scores for both AHI and ODI (ICC 0.958 and 0.986, respectively). In both the key measures, agreement between auto and the experts was higher than agreement between the two experts. Pearson's correlation between average of the two experts and auto was near-perfect, with ρ = 0.962. The IRR dropped for individual respiratory events both between the experts and between expert and auto. For all apneas, obstructive apneas, and oxygen desaturation events, the agreement between auto and the experts was close to between the experts. For central apneas, mixed apneas, hypopneas, and arousal, auto appeared to perform much worse than expert scorers. Even though the overall performance of auto for primary measures was excellent, the performance on these specific respiratory events can be improved further. Expert review of these events might play a significant role in improving these aspects.

### 4.3. Augmenting the sleep technologist

The primary goal of AI-based automatic scoring system is to empower sleep technologists by augmenting their capabilities. To achieve the highest levels of accuracy, reliability, and consistency, the experts must work in concert with AI. To fulfill this, it is important to understand the limitations of both manual and automatic scoring. Therefore, we carried out a thorough review of the scores by the experts at the clinic (expert 1). We did not optimize the review at this stage as the limitations of the auto scores were not known *a priori* and doing so would introduce bias into the process. Once the limitations were well understood, we proposed a data-driven approach to review the scores optimally.

Another reason why expert supervision is important is because AI-based systems can fail in ways that are counter-intuitive to humans. Under specific edge cases, AI can make mistakes that an expert would never make. For instance, on one occasion (outside the scope of the current study), due to an incorrect export of the PSG record, the data were incorrectly encoded in microvolts when in reality it was in volts. An error like this would be easily caught by the expert, although the AI failed to recognize it. Although the system was updated to handle such errors in the future, it is impossible to account for other such unforeseen edge cases.

### 4.4. Impact of a thorough review on staging

We already demonstrated that epoch-by-epoch staging was indistinguishable from experts. The only area where there is a scope to improve is latency. Following the thorough review, we observed that the majority of changes made by the experts was from N2 to N1, Wake to N1, and N2 to N3. These three changes accounted for nearly half of all the changes. This is in line with prior findings (10), where most of the confusion occurs between Wake, N2, and N1. The authors found that disagreement with stage N3 is almost entirely based on confusion with N2. Interestingly after review, the agreement with expert 1 did not change in terms of kappa, although there was an increase in accuracy from 79.59 to 82.2%. The agreement with expert 2 actually dropped from 79.59 to 78.41%. For the concordant epochs where both experts agree, it appears that the review had minimal to no effect (Figures 2B, C). Most epochs affected by the review probably did not have a clear classification. In a study analyzing inter-scorer variability (12), the authors observed that most of the variability is largely due to epochs that are difficult to classify and may not have a clear classification. This would explain why agreement following the review did not improve. As the automatic scores were already indistinguishable from the experts, the thorough review simply introduced a bias toward expert 1 while reducing agreement with expert 2.

While looking at derived sleep measures, agreement of automatic with expert was already better than between the experts for most measures, except for latency. Following review, we observed an increase in agreement for the latency measures. Therefore, we recommend a quick scan of the automatic scores with a focus on the first sleep and REM stages following wake as a strategy to optimally review sleep stages.

### 4.5. Impact of a thorough review on respiratory events

For respiratory events, automatic measurement of primary outcomes shows excellent agreement with experts, but there is significant scope for improvement for individual respiratory events. This is evident from the significant improvement in agreement for most respiratory events following the review. The only event that was negatively affected by the review was desaturation. ICC agreement for oxygen desaturation events is already almost perfect for automatic scoring. Therefore, our recommendation is to do a thorough review of respiratory events with a specific focus on arousals and apnea subtypes, while ignoring desaturation events.

Despite an additional review of sleep scoring manually by **expert 1** after completion of a first round of autoscoring, the average scoring time was reduced from 71 to 32 min. With the proposed optimal review strategy, we expect the scoring time to further reduce to ~15 min. We expect the automatic scoring system to have a significant impact on the economics and throughput of a sleep lab. A detailed analysis will be conducted and will be reported elsewhere.

### 4.6. Time motion study findings and impact on productivity

To the best of our knowledge, this is the first study to report potential productivity gains with the use of an automated software assessing PSG reports from patients with suspected sleep disorders. As highlighted earlier, with an estimated saving of 38.6 min per patient PSG report, this amounts to a total of 0.25 FTE savings per year based on an estimated load of 750 patients per year, assuming no improvements to the software over time and a stagnant patient workload. A further increase in productivity gains could be realized through improvements in software capability and accuracy, which would allow the sleep technologists to make fewer amendments during their manual checks on the automated PSG scorings, as well as a potential increase in patient workload due to the increase in suspected sleep disorders in the population. In the absence of any manual review, automatic scoring took only 42.7 s on average compared to 4,243 s for manual scoring, representing a 99.0% reduction in scoring time. With the rapid advancement in ML and increasing trust in AI systems, scoring might become an instantaneous or even a real-time process in the future.

## 5. Comparison with other commercial autoscoring systems

Most PSG data acquisition software includes some form of autoscoring system. Unfortunately, the performance of these systems has been less than satisfactory. These systems are mostly used to automatically identify oxygen desaturation events and leg movements. Most existing sleep-scoring solutions are based on rules or hand-engineered features and do not exploit recent advances in DL. DL-based algorithms in general perform better than rules or feature-based methods and generalize well beyond the training dataset. This is especially true when sufficiently large training datasets are available. To the best of our knowledge, EnsoSleep (Ensodata, WI, USA) is the only commercial solution cleared by the FDA that utilizes modern AI technologies. The most recent scoring performance of EnsoSleep is published in an abstract (29). The validation was carried out on 100 adult patients. The PSG records were scored by three RPSGTs and a 2/3 consensus was used as the ground truth. The automatic scoring showed very good agreement with the consensus scores. For respiratory events, a 30-s epoch was marked to contain an event if 2/3 of the experts agreed on the presence of it within the epoch. This was an unusual way of comparing respiratory scoring performance. Nonetheless, they demonstrated good performance for standard AHI thresholds. Commercial solutions that are well validated include Philips Somnolyzer (Philips Respironics, PA, USA), Morpheus 1 (WideMed, IL, USA), and Michele (Cerebra Health Inc., Winnipeg, Manitoba, Canada).

The most up-to-date validation (30) of the Philips Somnolyzer system was carried out on 97 records and scored by certified technologists from four sleep laboratories. The average correlation between expert-reviewed Somnolyzer scored AHI and experts was 0.930. For the hypopnea index, the pair-wise correlation varied between 0.570 and 0.940; for the central apnea index, the pair-wise correlation varied between 0.800 and 0.920; and for the obstructive apnea index, the pair-wise correlation varied between 0.790 and 0.880. For sleep staging, pair-wise ICC between expert reviewed Somnolyzer and the four experts varied between 0.30 and 0.60 for N1, 0.03 and 0.26 for N2, 0.10 and 0.24 for N3, 0.89 and 0.94 for REM, and 0.17 and 0.82 for the arousal index. Except for REM, differences were observed between automated and experts for the percentage of sleep in N1, N2, N3, and arousal index. Most metrics provided in the study were already reviewed by experts, and the scoring was carried out using more conservative 2007 AASM standards, which might also inflate agreement.

Validation for Michele's scoring system was carried out on 70 records (24) and scored by ten experts from five different sleep labs. The ICC agreement was 0.96 for AHI, 0.63 for central apnea, and 0.94 for obstructive apnea. For arousals, ICC was 0.39 for REM arousals and 0.83 for NREM arousals. ICC agreement for TST was 0.87, time in N1 was 0.56, time in N2 was 0.84, time in N3 was 0.47, time in REM was 0.64, sleep efficiency was 0.74, and REM latency was 0.55. An epoch-by-epoch agreement was not presented. An important limitation of the study was that a majority of the participants were healthy. The authors counted mixed apnea as obstructive. In addition, the records were scored as per the 2007 AASM standards, which could again inflate the agreement.

Morpheus 1 is the oldest of the three systems. The validation was carried out on 31 diagnostic PSG records and scored by two experts (25). Agreement between the two experts and Morpheus 1 was 77.7% with κ = 0.67 and 73.3% with κ = 0.61, while agreement between the two experts was 82.1% with κ = 0.73. The ICC for Morpheus 1 and expert 1 and expert 2 was 0.72 and 0.58, respectively, for the arousal index and 0.95 for both the experts for the respiratory disturbance index. The performance of Morpheus 1 was not on par with the experts.

Notwithstanding the fact that a direct comparison between Neurobit PSG with these systems is not possible given the datasets and the raters were different, Neurobit PSG consistently performed better across all measures for sleep staging despite scoring according to the more stringent 2012 AASM rules. Notably, for most key sleep and respiratory measurements, the agreement between auto and the experts was higher than that between the two experts.

## 6. Strengths and limitations

Some of the key strengths of the study are the relatively large and representative dataset, which was scored by trained sleep technologists independently. The two experts were from completely different continents to remove any potential training bias. The scoring was conducted in compliance with the latest AASM guidelines, which are more demanding as compared with previous standards. We provided an epoch-by-epoch comparison for staging including accuracy and kappa measures at both an aggregate and subject level. For respiratory events, we used a two-way random-effects model based on a single rater for absolute agreement to ensure the generalizability of our results beyond the raters involved in the study. The dataset used for benchmarking was completely independent of any training or testing data used in the development of the AI algorithm. This is important, as training, testing, and validating AI algorithms on the same dataset can introduce bias and significantly inflate the results. The study also provides productivity gain estimates through a thorough assessment using a time motion study. Despite all the strengths, the study has some limitations. There are only two sets of raters to estimate the baseline agreements. A larger number of raters could help make better IRR estimates for events that are less frequent, like mixed and central apnea and measures where agreements are known to be low between the experts like arousals and N1 duration. Another limitation of the study is that the scoring solution cannot be directly compared with existing commercial solutions. The AASM is currently working on a new platform to evaluate the performance of AI scoring packages. This is an excellent way to transparently evaluate commercial software solutions.

## 7. Conclusion

We benchmarked the performance of a new commercial sleep-scoring solution on a representative sample of patients with suspected sleep disorders. We demonstrated performance indistinguishable from experts in terms of staging and primary respiratory outcomes. Based on the review of the automatic scores by the experts, we observed the marginal utility of a thorough review of the staging. Although an extensive review of arousals, hypopnea, and apnea subtypes will improve scoring performance. We expect a significant benefit of AI-augmented sleep scoring in improving lab efficiency and scoring standardization, as well as potentially improving work productivity for sleep technologists in the healthcare setting.

## Data availability statement

The raw data supporting the conclusions of this article will be made available by the authors, without undue reservation.

## Ethics statement

The studies involving human participants were reviewed and approved by Singhealth Centralised Institutional Review Board. The patients/participants provided their written informed consent to participate in this study.

## Author contributions

The study was designed by HW, YM, BC, HO, YP, AP, and KK. AP, SBi, AA, and BC contributed to the analysis of data. YP was responsible for overseeing data collection and scoring at the hospital. YP, BC, AP, and KK were responsible for data handling and security. BC, AP, YM, HW, and SBh contributed to the write-up of the manuscript. All authors contributed to the article and approved the submitted version.
